# Cannabis Contaminants Limit Pharmacological Use of Cannabidiol

**DOI:** 10.3389/fphar.2020.571832

**Published:** 2020-09-11

**Authors:** Zackary Montoya, Matthieu Conroy, Brian D. Vanden Heuvel, Christopher S. Pauli, Sang-Hyuck Park

**Affiliations:** ^1^ Institute of Cannabis Research, Colorado State University–Pueblo, Pueblo, CO, United States; ^2^ Department of Biology, Colorado State University–Pueblo, Pueblo, CO, United States

**Keywords:** cannabis, cannabidiol, cannabis contaminants, hemp, phytocannabinoids

## Abstract

For nearly a century, *Cannabis* has been stigmatized and criminalized across the globe, but in recent years, there has been a growing interest in *Cannabis* due to the therapeutic potential of phytocannabinoids. With this emerging interest in *Cannabis*, concerns have arisen about the possible contaminations of hemp with pesticides, heavy metals, microbial pathogens, and carcinogenic compounds during the cultivation, manufacturing, and packaging processes. This is of particular concern for those turning to *Cannabis* for medicinal purposes, especially those with compromised immune systems. This review aims to provide types of contaminants and examples of *Cannabis* contamination using case studies that elucidate the medical consequences consumers risk when using adulterated *Cannabis* products. Thus, it is imperative to develop universal standards for cultivation and testing of products to protect those who consume *Cannabis*.

## Introduction

Phytocannabinoids have garnered global attention recently due to the therapeutic potentials in Parkinson’s disease (Chagas et al., 2014), Schizophrenia (McGuire et al., 2018), cancers (McGuire et al., 2018; Jeong et al., 2019; Sharafi et al., 2019), pain, anxiety, depression other neurological disorders (Marchetti, 2013) as well as the Food and Drug Administration (FDA) approval of Epidiolex for Dravet syndrome (Kaplan et al., 2017) and Lennox-Gauss Syndrome (Pauli et al., 2020). As of 2019, a total of 33 states, District of Columbia, Guam, Puerto Rico, and the U.S Virgin Island have approved *Cannabis* for medicinal purposes, 21 states are considering bills that would decriminalize it under legislative action. With recent legalization in Canada in 2019, more countries are beginning to question the rationale behind criminalizing *Cannabis* (Habibi and Hoffman, 2018). As interest in *Cannabis* expands throughout the globe, many issues have arisen concerning the lack of cultivation standards and overall quality control of *Cannabis* products. Recently the United States Pharmacopeia (USP) formed a *Cannabis* Expert Panel, which has evaluated specifications necessary to define key *Cannabis* quality attributes including limits for contaminants including pesticide residues, microbial levels, mycotoxins, and elemental contaminants based on toxicological considerations and aligned with the existing USP procedures for general tests and assays (Sarma et al., 2020). Aside from inaccuracy in labeling phytocannabinoid content, it has been reported that *Cannabis* and derived products are often contaminated by microbes, heavy metals, pesticides, carcinogens, and debris, which must be addressed to ensure the safety of consumers (**Table 1**) (Mcpartland and Mckernan, 2017; Dryburgh et al., 2018).

**Table 1:** List of *Cannabis* contaminants and sources, target/mechanism, and its respective risks to human health. Abbreviation: GABA: γ-amino butyric acid.

Contaminant		Source	Target/Mechanism	Health Risk	References
Microbes	*Aspergillus* Species	Soil/Environment	Pulmonary Infection	Aspergillosis (Keratitis, Onychomycosis)	(Gargani et al., 2011)
	*Penicillium* Species	Soil/Environment	Pulmonary Infection, Epidermal Invasion	Penicilliosis (Fever, Dry Cough, Skin Lesions)	(Gorai et al., 2019)
	*Fusarium Oxysporum*	Soil/Environment	Pulmonary Infection	Fusariosis (Fever, Neutropenia, Pneumonia, Sinusitis, Disseminated Disease in Immunocompromised)	(Dehal and Quimby, 2019)
	*Escherichia coli*	Soil/Handling	Enteric Infection, Brain Stem	Meningitis in Infants, Enteritis, Diarrhea	(Kim, 2016; Crofts et al., 2018; Valilis et al., 2018)
	*Salmonella*	Soil/Handling	Enteric Infection	Diarrhea, Vomiting, Fever, Enteritis	(Daley et al., 2013)
	*Clostridium*	Soil/Handling	Enteric Infection	Botulism (cranial nerve palsies, flaccid paralysis of voluntary muscles, respiratory compromise, death)	(Sobel, 2005)
Heavy Metals	Cadmium	Soil	Systemic	Periodontal Disease, Pancreatic Cancer, Diabetes, Itai-Itai Disease	(Kasuya et al., 1992; Buha et al., 2017; Tinkov et al., 2017; Browar et al., 2018)
	Lead	Soil	Systemic	Neurotoxic, Peripheral Neuropathy, Loss of Appetite and Weight, Chronic Fatigue	(Marchetti, 2013; Mason et al., 2014)
	Mercury	Soil	Systemic	Forgetfulness, Irritability, Restricted Visual Fields, Tremors, Paranoia	(Siegel et al., 1988)
Insecticides	Bifenazate	Applied During Growth	Unknown	Weight Loss and Gain	(Zarn and O’brien, 2017)
	Abamectin	Applied During Growth	GABA Mimetic Toxic Effects	Disruption of Synaptic Processes	(Da Silva et al., 2018)
Fungicides	Imazalil	Applied During Growth	Androgen Receptor Agonist and Endocrine Disruptor in Mammals	Abnormal Hormone Production	(Goetz et al., 2009)
	Myclobutanil	Applied During Growth	Inhibit Cholesterol Synthesis in Mammals	Not Known for Humans	(Hester et al., 2006; Berenstein et al., 2017)
Plant Growth Regulator	Daminozide	Applied During Growth	Unknown	Considered a Human Carcinogen	Neff and Goldman, 2005)
	Paclobutraxol	Applied During Growth	Disrupt Neurotransmitter Levels in Mammals	Not Known for Humans	(Li et al., 2012; Xu and Yang, 2020)
Polycyclic Aromatic Hydrocarbons	PAHs	Ubiquitous Environmental Toxins	DNA Adducts, Histone Alteration, DNA Methylation	Carcinogenic	(Abdel-Shafy and Mansour, 2016)

These contaminants are imminent threats that directly impact public health and wellness, particularly to the immunocompromised and pediatric patients who take *Cannabis* products as a treatment for numerous human disorders including cancer patients and those suffering from epileptic seizures (Ruchlemer et al., 2015). To increase public awareness, we provide examples of contamination, its medical consequences reported in clinical research, and then suggest that each risk category be analyzed for best practices to limit exposure of contaminants to the consumer. We recommend hemp producers, manufacturers, medical professionals, and legislators recognize this risk and establish regulatory measures to educate the public and lessen the adverse effects caused by the contaminants in *Cannabis*, particularly in cannabidiol (CBD)-based products.

## Labeling Inaccuracy

Mislabeling of phytocannabinoid profiles in CBD products is one of the major concerns to consumers (Hazekamp, 2018). Inaccurate reporting of the cannabinoid content risks exposing medicinal users to phytocannabinoids of which they have no intent to consume, namely Δ-9-tetrahydracannabinol (THC) (Corroon et al., 2020). This is of particular concern within pediatric patients, as THC intoxication has been shown to alter development of white matter in the brain (Gruber et al., 2014), as well as affect cognitive functioning (Crean et al., 2011; Zamberletti et al., 2015), and learning and memory within adolescents (Wang et al., 2013).

Despite a 15% allowable reporting variance of the phytocannabinoid content on CBD product labels (Herod et al., 2018), measured contents often exceed this range. For example, a recent study shows that 69% of 84 CBD products purchased from 31 American online retailers were inaccurately labeled for CBD, 26% were overlabeled whereas 42% were underlabeled for CBD concentration (Bonn-Miller et al., 2017). Additionally, 64% of 14 CBD products sold in the European Union (EU) market presented different cannabinoid profiles from the declared amount (Pavlovic et al., 2018). Inaccuracies are also found on labels of hemp-type *Cannabis* sold in the Netherlands with measured THC and CBD deviating from label claims by 8%–99% in CBD oil samples obtained from patients (Hazekamp, 2018). In Germany, an analysis of 67 CBD product samples it was found that 25% of samples were contaminated with residual THC above the lowest level of observable effects, or the lowest level that is known to cause physiological effects in humans (2.5 mg/day) (Lachenmeier et al., 2019). A recent analysis of 25 CBD oil products purchased in Mississippi, of the 25 products, only 3 were within ±20% of label claim, 15 were below the stated claim for CBD, 2 exceed these claims by more than 50%, and THC content for 3 products exceeded the 0.3% legal limit (Gurley et al., 2020). There are also concerns for edible *Cannabis* products (e.g., gummies, cookies, etc.) containing under and over reported phytocannabinoid content, specifically THC (Vandrey et al., 2015). In states where *Cannabis* is legal for recreation, these edible products are tested for overall THC potency in addition to dose specific potency of THC to be sure these products stays under 100 mg total THC with no more than 10 mg of THC per dose (Blake and Nahtigal, 2019). However, in hemp, there is no regulating body overseeing this testing, therefore the responsibility to test CBD edibles is left to each product manufacturer to ensure compliance of cannabinoid content limits, which is often neglected (Blake and Nahtigal, 2019). While currently CBD and THC are the only cannabinoids required to be labeled, it may be beneficial to include the profile of acidic forms of THC and CBD and some representative minor cannabinoids such as cannabigerol (CBG), cannabichromene (CBC), or possibly some of the short chain versions of these referred to as Varin cannabinoids on these labels. These minor cannabinoids are shown to have some therapeutic effects that could be enhanced in combination with other major cannabinoids (Deiana, 2017).

## Microbial Contamination

*Cannabis* is associated with various types of microbes including molds that have been shown to harm immunocompromised patients, as well as bacteria and viruses that have the potential of causing harm to humans. A recent metagenomics study on 15 medicinal *Cannabis* plants shows that *Cannabis* is associated with a wide range of epiphytic and endophytic microbial communities including several toxigenic bacterial and fungal species (Mckernan et al., 2016). While most of the microbes found to be in association with *Cannabis* are likely beneficial to the plant in some way or phytopathogens, several bacterial species have been identified that could be opportunistic pathogens in humans (Mckernan et al., 2016). While there are currently no reports of bacterial infection caused by contaminated *Cannabi*s, several examples of fungal contamination, namely *Aspergillus* sp., are found in the literature and pose a threat to human health (Mckernan et al., 2016). In this section, the authors will introduce some of the possible human pathogenic microbial species and their relevant case studies.

### Fungal Contaminants

Previous studies have identified several fungal organisms in dispensary-produced *Cannabis* including *Penicillium* sp. (*P. paxilli*, *P. citrinum*, *P. commune*, *P. chrysogenum*, *P. corylophilum*, *P. citrinum*, and *P. steckii*), *Aspergillus* sp. (*A. terreus*, *A. niger*, *A. flavus*, *A. versicolor*, *A. ostianus*, and *A. sydowii*), and *Fusarium* sp. (*F. oxysporum*) (Mcpartland and Hillig, 2004; Mckernan et al., 2016). Both *Penicillium* sp. and *Aspergillus* sp. have been known to produce aflatoxins (e.g., aflatoxin B1) while *Fusarium* species produce other mycotoxins such as fumonisin (Daley et al., 2013; Punja et al., 2019). *Cannabis* infected with *Aspergillus*, *Penicilium*, or *Fusarium* can severely affect human health as these toxins can all be carcinogenic, hepatotoxic, neurotoxic or nephrotoxic (Pitt et al., 2000; Marasas et al., 2004; Kamle et al., 2019). The toxicological action depends on various factors including the mode of exposure and the susceptibility of the infected individual, which the immunocompromised patients have the highest risk of infection (Pitt et al., 2000). In general, these toxins are carcinogenic as they commonly interact with guanine moieties in DNA forming a variety of DNA adducts which often leads to deterioration of the liver (Pitt et al., 2000).

#### Case Study 1

Penicilliosis, a fungal infection due to *Penicillium* species, is rare in immunocompetent people but is commonly the cause of death for human immunodeficiency virus (HIV) positive and other immunocompromised patients (Le et al., 2020). Currently, no reports of Penicilliosis caused by *Cannabis* are found in the literature; however, immunocompromised individuals should be cautious using *Cannabis* products as several species known to cause this condition have been found in *Cannabis* flowers and products (Mcpartland and Hillig, 2004; Mckernan et al., 2016).

#### Case Study 2

*Aspergillus* species are the most common fungi to cause invasive infection in the immunocompromised. This is concerning as *Aspergillus* infected *Cannabis* has been previously directly linked to human disease (Gargani et al., 2011). A case study showed that a patient with lung cancer used illicitly obtained *Cannabis* as an antiemetic agent during chemotherapy and developed invasive pulmonary aspergillosis that caused death in 19 days after diagnosis (Sutton et al., 1986). Many of the recent metagenomic studies of *Cannabis* show that *Aspergillus* species are still pervasive in *Cannabis* which may pose a considerable risk to the consumer, especially the immunocompromised (Mckernan et al., 2016).

#### Case Study 3

*Fusarium* species are common environmental fungi, capable of causing infections in both animals and plants (Kamle et al., 2019). Humans infected by *Fusarium* present with a wide range of symptoms including fever, neutropenia, pneumonia, sinusitis, or disseminated disease in some immunocompromised patients (Dehal and Quimby, 2019). *Fusarium* can cause a pulmonary infection that could result from the inhalation of conidia, a spore produced by these asexual fungi, that is consistent with the following case (Sreeram et al., 2017). A recent case study in an immunocompromised patient with Acute Myeloid Leukemia, who developed invasive disseminated Fusariosis, has proven infection by this fungus is fatal (Dehal and Quimby, 2019). The patient initially presented with painless lesions on her arms and legs, that darkened, grew, and spread to her trunk and all extremities. The patient elected to discontinue treatment and passed two weeks after transitioning to hospice care (Dehal and Quimby, 2019). There are limited case studies demonstrating *Cannabis* causing Fusariosis; however, there are a plethora of studies that have found Fusarium to be in direct relationship with *Cannabis* (Mcpartland and Hillig, 2004; Punja et al., 2019). In fact, starting in the late 1970’s through the 1980’s, *F. oxysporum* was physically distributed across the United States to combat illegal *Cannabis* farming (Mcpartland and Hillig, 2004). While this was intended as a short-term biological control, it has inevitably caused this organism to continually infect legal hemp and *Cannabis* farms today, which may negatively impact the quality of *Cannabis* grown in legal markets.

In addition to pathogenesis in humans by these fungi, *Penicillium*, *Aspergillus*, and *Fusarium* sp. are known to produce both aflatoxins and mycotoxins that become especially problematic while drying and storing *Cannabis* products in humid environments (Mcpartland and Cubeta, 1997; Mcpartland and Mckernan, 2017). Several *Cannabis* drying strategies, such as sweat curing, make samples more susceptible to contamination from various types of *Aspergillus* because of relatively high water activity inside the stacked plant materials (Mcpartland and Mckernan, 2017). Sweat curing is not as commonly practiced today; although, there have still been recent reports of unacceptable levels of fungal spores in products grown in both indoor and outdoor facilities (Mcpartland and Mckernan, 2017). This indicates some current methods of cultivation and curing still leave the plant susceptible to fungal infection (Martyny et al., 2013). As such, standard testing procedures of fungal mycotoxins in *Cannabis* for both the hemp- and drug-type markets must be developed and are imperative to best protect the consumer, especially those with a compromised immune system using *Cannabis* as a therapy.

### Bacterial Contaminants

Bacterial contamination is less of a direct health threat to *Cannabis* users than fungus and molds, but there have been potentially pathogenic species identified in a few recent studies (Mckernan et al., 2016; Mcpartland and Mckernan, 2017; Sandler et al., 2019). A study of five *Cannabis* cultivars had shown that most species of bacteria were identified from samples of endorhiza-, rhizosphere-, and bulk soil-associated microbiomes more so than from other regions of the plant. These bacteria contaminate include various species of *Pseudomonas, Cellvibrio, Oxalobacteraceae, Xanthomonadaceae, Actinomycetales*, and *Sphingobacteriales* in the examined microbiomes (Winston et al., 2014). Another study shows a variety of potential human pathogens, including *Acinetobacter baumannii, Escherichia coli, Pseudomonas aeruginosa, Ralstonia pickettii, Salmonella enterica, Stenotrophomonas maltophilia*, and *Clostridium botulinum*, in the flowers of medicinal *Cannabis* plants grown at indoor facilities in Massachusetts, Maine and Rhode Island (Mckernan et al., 2016). Endophytic bacterial taxa have also been identified that may provide fungal resistance and other fitness-related traits to *Cannabis* through secondary metabolite production, some of which could be used in growth promotion and/or in biological control designed experiments (Scott et al., 2018). Although some bacteria have been shown to be beneficial to cultivation, the possible pathogenic species that have been associated with *Cannabis* are of greater concern, specifically the risk these species pose to consumers.

While dozens of bacterial species found to be present in *Cannabis* plants, *E.coli*, *Salmonella*, and *Clostridium* are a few common potential human pathogenic species shown to be associated with *Cannabis* (Mckernan et al., 2016). *Escherichia coli* infection has potential to cause a wide range of diseases depending on the strain encountered, including meningitis in infants, enteritis, and diarrhea (Kim, 2016; Crofts et al., 2018; Valilis et al., 2018). Exposure to *Salmonella* can cause bacterial infection with symptoms including diarrhea, vomiting, fever, and enteritis (Daley et al., 2013). Clostridium can cause botulism, a rare disease with symptoms including cranial nerve palsies and flaccid paralysis of voluntary muscles, with potential progression to respiratory illness and death (Sobel, 2005).

There are also concerns for the contamination of *Cannabis* food products by potentially harmful bacteria including *Listeria* (Mckernan et al., 2018). *Listeria* sp. have been shown to be opportunistic pathogens that most commonly cause food poisoning or Listeriosis; however, if infecting the central nervous system, these bacterium can induce encephalitis or mimic idiopathic inflammatory demyelinating disease (Morgand et al., 2018). Though the presence of these bacteria have been reported as highly prevalent in *Cannabis* (Mcpartland and Mckernan, 2017), current literature does not reflect any opportunistic infection caused by use of bacterial-contaminated *Cannabis* products. Still, the presence of human pathogenic bacteria on *Cannabis* presents a possible risk to the consumer especially the immunocompromised, therefore ways to limit bacterial contamination should be explored.

### Viral Contaminants

Our literature searches yielded no reports of human pathogenic viral contamination of *Cannabis* but other crops have shown contamination by various noroviruses, rotaviruses, and enteroviruses causing enteric diseases in humans (Bouwknegt et al., 2015; Pérez-Moreno et al., 2019). Viruses found to be associated with *Cannabis* are purely plant pathogens, and it is not assumed that these could cause human related diseases (Mcpartland and Mckernan, 2017). However, human handling in this industry is frequent, and it is possible that the product could be contaminated with a human pathogen through contact. While no reported cases of viral infection caused by *Cannabis* use are found in the literature, this is not a largely explored area of research and should be considered in future studies. It is possible that human viral pathogens will be identified through further metagenomic studies of *Cannabis* and until the risk of disease can be ruled out, viral contamination should be considered possible.

## Heavy Metal Contamination

A variety of heavy metals have been found in *Cannabis* plants and products made with *Cannabis* (e.g. tinctures and oils), including cadmium, lead, magnesium, copper, and mercury (Siegel et al., 1988; Busse et al., 2008; Mcpartland and Mckernan, 2017; Dryburgh et al., 2018; Gauvin et al., 2018). *Cannabis* plants have been shown to hyperaccumulate and incorporate these metals into tissues throughout the plant and have been previously explored for their ability to bioremediate contaminated soils (Mcpartland and Mckernan, 2017). Most heavy metals have low biodegradability, which allows them to bioaccumulate up the food chain and persist in the body long-term causing a wide range of health problems (Vardhan et al., 2019). Furthermore, many heavy metals have been shown to have fatal effects in humans when exposed both acutely or chronically, causing a plethora of diseases, such as, cancers and neurological disorders (Daley et al., 2013). While Colorado and California do require heavy metal testing in *Cannabis*, without similar requirements in place to test for such heavy metal contamination in hemp and CBD products, many people are at risk of exposure to toxic levels of heavy metals. At the greatest risk for detrimental effects from heavy metal contamination are those using CBD as a medical treatment including children suffering from pediatric epilepsy, and the various conditions leading to compromised immune systems. Thus, the authors aim to identify medical consequences of exposure to three major heavy metal contaminants found in *Cannabis* (i.e., cadmium, lead, and mercury) with a corresponding case study for each.

### Cadmium

Cadmium is a soft, bluish white heavy metal that is mainly obtained from zinc ore processing, has a long biological half-life of 14 to 24 years, and bioaccumulates in the human body when chronically exposed (Pappas, 2011). The impact of exposure to cadmium containing products and cadmium containing fertilizers on humans remains a major concern (Tellez-Plaza et al., 2013). Leafy and root vegetables, grains, and tobacco bioconcentrate cadmium from the soil resulting in exposure through diet and smoking (Tellez-Plaza et al., 2013). Cadmium is generally found in higher levels in urine, blood, fat, and lung tissues of tobacco smokers which correlates with length of time as a smoker (Pappas, 2011). It has also been shown that application of phosphate fertilizers targeted for *Cannabis* growth increases the uptake of cadmium by *Cannabis* when grown in cadmium contaminated soils though the mechanism is not yet clear (Singani and Ahmadi, 2012). Currently, no cases of cadmium contaminated *Cannabis* causing health problems are found in the literature; however, there are several diseases that have been associated with exposure to cadmium through smoking and diet including periodontal disease (Browar et al., 2018), pancreatic cancer (Buha et al., 2017), and diabetes (Tinkov et al., 2017). Most severely, chronic exposure causes Itai-Itai disease, which is characterized by intense bone pain, a disrupted gait, and numbness in all extremities (Kasuya et al., 1992). *Cannabis* products are not likely to be contaminated enough to cause disease as severe as Itai-Itai disease, but because *Cannabis* can hyperaccumulate cadmium, it should still be considered hazardous and tested for in *Cannabis* products.

### Lead

Lead is a silver to dark gray, soft, malleable, corrosion resistant heavy metal and is one of the earliest metals discovered (Flora et al., 2012). Lead has been used in automobile, paint, ceramic and plastic manufacturing and because lead is nonbiodegradable, it persists in the environment (Flora et al., 2012). Lead can have a variable biological half-life from 30 days for lead in the blood and up to 30 years for lead deposited into bone, which is usually a sign of chronic exposure (Mason et al., 2014). Not only has lead been found in *Cannabis* but the uptake of lead has been shown to increase in *Cannabis* grown in contaminated soils (Singani and Ahmadi, 2012), especially in contaminated urban environments (Entwistle et al., 2019). Once lead enters the body, it can interact with almost every organ; however, its effects on the central nervous system are the most severe (Mason et al., 2014). Lead acts as a calcium analog interfering with ion channels of mammalian neurons (Marchetti, 2013). It has also been observed that lead is a potent reversible and selective blocker of voltage-dependent calcium channels even at low concentrations in human neurons (Marchetti, 2013; Mason et al., 2014). Lead contamination of *Cannabis* products sold in legal markets will likely be due to the cultivation of *Cannabis* in contaminated soils, but lead has been deliberately added to *Cannabis* as well. This is highlighted by an incident of massive lead poisoning in Leipzig Germany where lead was intentionally added to *Cannabis* attempting to increase its mass and in turn its street value, which caused 35 people to be treated for blood lead levels up to 1,063 µg/L (Busse et al., 2008). Symptoms experienced by these patients included nausea, acute colic, formation of a lead seem along the dental margin, peripheral neuropathy, loss of appetite and weight, as well as chronic fatigue and exhaustion (Busse et al., 2008). An additional 597 *Cannabis* users in the area of Leipzig reported for a screening program initiated by the local health office of which 27% of patients were found to have levels exceeding human biomonitoring values (above 250 µg/L for men and 150 µg/L for premenopausal/fertile women) also necessitating treatment (Busse et al., 2008). Lead contamination of *Cannabis* products should be avoided and considered a major health risk to all users.

### Mercury

Mercury is another hazardous heavy metal found in *Cannabis*, silver in color, the only liquid metal at standard temperature (0° C or 273.15 K) and pressure (1 atm, 101.3kPa, or 760 mmHg) in its elemental form (Hg); however, it is also frequently found in organomercury compounds such as methylmercury which is considered highly poisonous and deleterious to humans when ingested (Hong et al., 2012). In this organomercury form, methylmercury bioaccumulates in the human body, and has a biological half-life of up to 80 days for methylmercury that does not cross the blood brain barrier (Jo et al., 2015). Although after crossing the blood brain barrier, methylmercury persists for decades in the brain after the cessation of exposure (Björkman et al., 2007). Mercury has been found at up to 440 ng/g of dry mass of *Cannabis* grown in volcanic soils in Hawaii (Mcpartland and Mckernan, 2017). This is cause for concern as smoking *Cannabis* products greatly increases the risk of heavy metal toxicity with mercury being absorbed 10 times more efficiently by the lungs than the gut (Mcpartland and Mckernan, 2017). Chronic human exposure to mercury vapor results in a variety of symptoms, largely neurological, including forgetfulness, irritability, restricted visual fields, tremors, and paranoia (Siegel et al., 1988). Considering the health risks, it is in the interest to all *Cannabis* users that mercury contamination be avoided as it would have detrimental effects on all exposed.

## Pesticide Contamination

While many claim *Cannabis* is naturally a pest resistant crop (Park et al., 2019; Mckernan et al., 2020), there is still abundant use of various types of pesticides to provide protection, including insecticides, fungicides, and plant growth regulators (Mcpartland and Mckernan, 2017; Sandler et al., 2019). Unlike most crops that are grown in the United States, there are no federal guidelines provided by the Environmental Protection Agency (United States Environmental Protection Agency, 2019) as to which pesticides or how much should be used on *Cannabis* (Seltenrich, 2019). With the recent federal legalization of hemp in the U.S. under the 2016 and 2019 Farm Bills, a limited number of pesticides have been approved for use with hemp or *Cannabis* plants in the U.S. (United States Environmental Protection Agency, 2019). Previous to the release of this list, a lack of regulation led to the widespread use of hazardous pesticides including bifenazate, myclobutanil, and daminozide, as well as several others intended for ornamental plants and which are not approved for human consumption (Mcpartland and Mckernan, 2017). For example, the pesticide contents of 26 *Cannabis* samples obtained from Washington dispensaries were investigated, and 84% of the *Cannabis* samples analyzed were found to contain up to 24 agents of insecticides, miticides, fungicides, insecticidal synergists, and plant growth regulators (Russo, 2016). Also in 2016, it was found that 49% of *Cannabis* samples obtained from California dispensaries contained pesticides that are purely for ornamental plants including abamectin and bifenazate (Mcpartland and Mckernan, 2017). In 2016, it was also shown that Guardian pesticides, which were marketed as all natural containing only safe to consume chemicals like cinnamon oil and citric acid, did in fact contain abamectin (Mcpartland and Mckernan, 2017). Furthermore, it has been shown that 69% of the pesticides used in cultivation stay in *Cannabis* during smoking and can create toxic pyrolytic side products, suggesting that pesticide contaminated Cannabis may pose a significant toxicological threat to its users (Sullivan et al., 2013).

As many of these pesticides are lipophilic, they are soluble in the solvents used for extraction of cannabinoids, including CBD oils and other products using extracted cannabinoids. Naturally, this leads to concerns about contamination of *Cannabis* with pesticides and the potential health risks that would accompany concentrating these pesticides in an extract. The authors provide examples of a few compounds found in *Cannabis* from each class of pesticides and the potential health risks posed by each. While it is well beyond the scope of this paper to review all types of pesticides used to treat *Cannabis*, it is clear that pesticides associated with *Cannabis* and their individual health risks should be considered as important to growers and user alike. For the health of consumers, particularly those with compromised immune systems utilizing *Cannabis* for its therapeutic properties, it is imperative that a standard protocol continue to be developed for the safe use and testing of pesticides in *Cannabis*.

### Insecticides

Bifenazate (propan-2-yl *N*-(2-methoxy-5-phenylanilino) carbamate) and abamectin [(1’R,2R,3S,4’S,6S,8’R,10’E,12’S,13’S,14’E,16’E,20’R,21’R,24’S)-2-butan-2-yl-21’,24’-dihydroxy-12’-[(2R,4S,5S,6S)-5-[(2S,4S,5S,6S)-5-hydroxy-4-methoxy-6-methyloxan-2-yl]oxy-4-methoxy-6-methyloxan-2-yl]oxy-3,11’,13’,22’-tetramethylspiro [2,3-dihydropyran-6,6’-3,7,19-trioxatetracyclo[15.6.1.14,8.020,24]pentacosa-10,14,16,22-tetraene]-2’-one)] are two commonly identified insecticides found on *Cannabis* products that are known to be harmful to mammals (Radi et al., 2020). Bifenazate, a spider miticide, is not considered to be acutely toxic, though is considered to be toxic when chronically exposed to mammals (European Food Safety Authority, 2017). In animal feeding studies, weight gain in males and weight loss in females were reported in response to chronic exposures of bifenazate in their diets (Zarn and O’brien, 2017). Furthermore, bifenazate has only been approved for use on ornamental plants in the U.S., so the EPA has not released any information regarding human mutagenicity for this compound. Abamectin, a macrocyclic lactone, is generally considered safe with toxicity arising only after ingestion of large quantities and is approved for edible plants (Da Silva et al., 2018). Although the exact mechanisms remain unclear, there is evidence that macrocyclic lactones in large doses may pass through the blood-brain barrier to produce γ-amino butyric acid -mimetic (GABA) toxicity-like effects (Da Silva et al., 2018). Current gaps in the knowledge of the long-term effects of these compounds still exist, but cell culture and animal studies continue to shed new light on the overall health impacts of these compounds. Until these compounds are shown to be harmless when inhaled or ingested, their application to *Cannabis* should be limited or ceased entirely to best protect the consumers health.

### Fungicides

Several fungicides have been reported in samples of *Cannabis* all over the world including known endocrine disruptors and hepatoxic compounds like imazalil (1-[2-(2,4-dichlorophenyl)-2-prop-2-enoxyethyl] imidazole) and myclobutanil (2-(4-chlorophenyl)-2-(1,2,4-triazol-1-ylmethyl) hexanenitrile), respectively (Mcpartland and Mckernan, 2017; Sandler et al., 2019). These fungicides are often found in higher concentrations in samples obtained from indoor grow facilities than outdoor operations (Mcpartland and Mckernan, 2017). As many medicinal *Cannabis* cultivators are now using indoor facilities to cultivate hemp for CBD products year-round, this is a cause for concern in both the hemp- and drug-type markets.

Imazalil also known as enilconazole and myclobutanil have both been used to prevent fungal infection in *Cannabis* (Mcpartland and Mckernan, 2017). Imazalil, a systemic fungicide used to control powdery mildew and other mold or fungal infections in crop plants, has been shown to be an androgen receptor agonist and endocrine disruptor in mammals (Goetz et al., 2009). It can cause detrimental mutations in genes controlling cholesterol metabolism and androgen conversion to estrogen that carry on and persist into the following generations in mammals (Jin et al., 2018; Jin et al., 2019). Myclobutanil is an inhibitor of ergosterol production in fungus which is essential for the formation of fungal cell walls (Hester et al., 2006). In addition to being detrimental to fungi, myclobutanil can also inhibit cholesterol synthesis in mammals at high doses (Hester et al., 2006; Berenstein et al., 2017). Neither of these compounds have been extensively studied in humans, but what is known about their effects on mammalian systems is cause for concern. These fungicides should not be considered safe to use for any *Cannabis* cultivation, and their application should be avoided to protect the health of the consumer.

## Plant Growth Regulators

Plant growth regulators are also commonly found in *Cannabis*, including carcinogens and compounds that have been shown to be detrimental to mammals. Daminozide (4-(2,2-dimethylhydrazinyl)-4-oxobutanoic acid) and paclobutraxol ((2*R*,3*R*)-1-(4-chlorophenyl)-4,4-dimethyl-2-(1,2,4-triazol-1-yl)pentan-3-ol) are two plant growth regulators pervasively found in *Cannabis*. Daminozide is used to delay the ripening of fruits and is considered relatively nontoxic unless consumed at very high doses; however, it is still considered a human carcinogen by EPA (Neff and Goldman, 2005). This may be a greater concern to the farmers and cultivators than the end user, although little is known about the chronic exposure to daminozide over long periods of time. Paclobutraxol is a plant growth retardant that inhibits the biosynthesis of the plant hormone gibberellin which is responsible for shoot elongation (Rademacher, 2000). Paclobutraxol has been shown to have detrimental effects on development in several aquatic species (Li et al., 2012), and also to disrupt neurotransmitter levels in mice (Xu and Yang, 2020). Considering little is known about the human health consequences of chronic exposure to plant growth regulators, the use of these compounds in *Cannabis* cultivation should be regulated with the health of the consumer in mind.

## Polycyclic Aromatic Hydrocarbons

Polycyclic aromatic hydrocarbons (PAH) are ubiquitous environmental pollutants usually generated by the incomplete combustion of organic materials (e.g. oil, coal, and wood) (Abdel-Shafy and Mansour, 2016). They are found in some CBD oils and may come either from uptake by the plant during growth or from contaminated carrier oils during product preparation (Večerka, 2018). Excessive PAH content in CBD oils can be attributed to the smoke from nearby forest fires or from drying *Cannabis* with propane heaters (Zelinkova and Wenzl, 2015). Over 100 types of PAHs exist and some of the most studied and well characterized (*i.e*., benzo anthracene, chrysene, benzo fluoranthene, benzo pyrene) are known to be hazardous, deemed as carcinogens, and can found in *Cannabis* products worldwide. In EU, 20 out of 29 tested CBD oil brands were shown to have PAH levels higher than the legislative limits of 20 mg/kg (White, 2019). High levels of PAHs in CBD oil are likely to cause DNA methylation, DNA adducts, and alteration of histone methylation which can lead to immunosuppression (Abdel-Shafy and Mansour, 2016). The elimination of PAHs in the environment is most studied in biological systems through multi-step metabolic pathways, primarily mixed-function oxidase systems, but they can degrade through oxidation reactions in the environment (Abdel-Shafy and Mansour, 2016). The extent to how any given PAH is eliminated is highly dependent on its unique physical and chemical properties (Abdel-Shafy and Mansour, 2016). While it may be impossible to eliminate PAHs in *Cannabis* products due to the ubiquitous nature of PAHs in the environment and the risk of producing these compounds when smoking *Cannabis*, consumer exposure can be reduced by addressing the sources of contamination and avoiding growing *Cannabis* in heavily industrialized areas (Jett et al., 2018).

## Other Foreign Matters

Other debris such as metal fragments, hairs, dusts, machine oils, or insect parts can be found in some CBD oil products as is seen in other foods or food products (Dryburgh et al., 2018). The FDA considers these foreign contaminants a negligible health hazard but clearly this needs to be addressed by manufactures to develop high-quality control standards required to limit and minimize any foreign matter contamination.

## Discussion

With the recent legalization of *Cannabis* in many states of the U.S., there have been several state regulatory commissions put into place that address the issue of quality control in terms of contaminants and cannabinoid profile. However, the testing requirements do vary from state to state in terms of the minimum number of contaminants that must be tested for, and only 15 states currently have a regulatory commission in place. For the safety and welfare of all users, both medicinal and recreational, there is a necessity for a standardized set of guidelines for cultivation and testing of *Cannabis* products. There is currently only one set of guidelines called *Recommendations for Regulators — Cannabis Operations* that provides detailed set of recommended instructions on cultivation, packaging, testing and dispensing of *Cannabis* products including both THC and CBD products, which has proven invaluable for ensuring the safe cultivation of *Cannabis* (American Herbal Products Association, 2016). While these are a great set of guidelines, a more comprehensive understanding of the contamination of *Cannabis* products is necessary to appropriately eliminate the possible deleterious health effects contaminates may cause. Unfortunately, the classification of *Cannabis* as a schedule 1 drug federally makes the development and implementation of nationwide standards impossible at the moment, which if left unchanged, could lead to significant health complications in those turning to *Cannabis* for its medicinal properties.

## Author Contributions

S-HP and BVH conceived the review idea. MC and ZM wrote the initial draft. S-HP and CP reviewed and edited the manuscript. All authors contributed to the article and approved the submitted version.

## Funding

This study was funded by the Institute of Cannabis Research (ICR).

## Conflict of Interest

The authors declare that the research was conducted in the absence of any commercial or financial relationships that could be construed as a potential conflict of interest.
